# The Targeting of Native Proteins to the Endoplasmic Reticulum-Associated Degradation (ERAD) Pathway: An Expanding Repertoire of Regulated Substrates

**DOI:** 10.3390/biom11081185

**Published:** 2021-08-11

**Authors:** Deepa Kumari, Jeffrey L. Brodsky

**Affiliations:** Department of Biological Sciences, University of Pittsburgh, A320 Langley Hall, Fifth & Ruskin Ave, Pittsburgh, PA 15260, USA; DEK89@pitt.edu

**Keywords:** ERAD, molecular chaperones, ubiquitin, proteasome, protein quality control

## Abstract

All proteins are subject to quality control processes during or soon after their synthesis, and these cellular quality control pathways play critical roles in maintaining homeostasis in the cell and in organism health. Protein quality control is particularly vital for those polypeptides that enter the endoplasmic reticulum (ER). Approximately one-quarter to one-third of all proteins synthesized in eukaryotic cells access the ER because they are destined for transport to the extracellular space, because they represent integral membrane proteins, or because they reside within one of the many compartments of the secretory pathway. However, proteins that mature inefficiently are subject to ER-associated degradation (ERAD), a multi-step pathway involving the chaperone-mediated selection, ubiquitination, and extraction (or “retrotranslocation”) of protein substrates from the ER. Ultimately, these substrates are degraded by the cytosolic proteasome. Interestingly, there is an increasing number of native enzymes and metabolite and solute transporters that are also targeted for ERAD. While some of these proteins may transiently misfold, the ERAD pathway also provides a route to rapidly and quantitatively downregulate the levels and thus the activities of a variety of proteins that mature or reside in the ER.

Proteins play myriad roles in cells, and the acquisition of their final folded states and structural integrity are imperative for function. However, the expression of misfolded or non-native protein structures can lead to a plethora of diseases [1,2,3,4]. To destroy these misfolded and potentially toxic proteins, eukaryotic cells survey structural integrity at multiple checkpoints as a protein matures. The factors that survey these nascent proteins, attempt to repair misfolded species, facilitate maturation, target misfolded substrates for degradation, and trigger cellular responses to off-set proteotoxicity collectively maintain protein homeostasis, or “proteostasis”, in the cell.

Between a quarter to one-third of all nascent proteins enter the endoplasmic reticulum (ER) during or soon after synthesis. These diverse substrates then fold, become post-translationally modified, assemble into protein complexes, and/or insert into the lipid bilayer in the membrane [5,6,7]. As a nascent protein undergoes these events, it is then subject to surveillance by several ER quality-control pathways. One of these pathways was named endoplasmic-reticulum-associated degradation (ERAD) [8,9], a pathway requiring a complex collection of factors that help eliminate aberrant proteins that pose a threat to the ER and consequently cellular homeostasis [10,11,12,13]. Therefore, this protein quality-control pathway clears the secretory pathway of potentially toxic proteins. During ERAD, the aberrant protein is first recognized by chaperones, and after selection, it is then retrotranslocated into the cytosol and ubiquitinated by one of a collection of E3 ubiquitin ligases. The ERAD substrate is finally transported to and degraded by the cytoplasmic proteasome (Figure 1).

Although ERAD was first discovered and was initially thought to primarily target misfolded proteins [8,15,16,17,18,19], the ERAD pathway also regulates steady-state levels and thus, the activities of several apparently natively folded proteins (see Table 1 for select examples) (also see [20,21,22]). In retrospect, this is not surprising since there are >7000 proteins that interact with the ER at some point during biogenesis in human cells [23], and these proteins support myriad physiological processes, many of which are regulated. In fact, one regulated wild-type substrate, HMG CoA-reductase (see below), appears to become unfolded in response to a metabolic signal/lipid, which destabilizes and targets the enzyme for ERAD [24]. It is likely that a similar scenario is evident for other regulated, functioning enzymes. In effect, the rapid degradation of these proteins represents the best and most complete way to downregulate function. Overall, ERAD maintains quality control—i.e., the degradation of misfolded and improperly modified proteins—as well as quantity control in the secretory pathway.

In the remaining sections of this article, we provide a brief overview of the classes of natively folded proteins that are ultimately regulated by ERAD. These substrates are enriched for proteins that are linked to lipid metabolism but also include those that are linked to metabolite and solute transport. To better focus on the active enzymes and transporters regulated by ERAD, we will exclude a discussion of ERAD substrates that are misfolded, represent unassembled units of multimeric proteins, or are regulated by pathogens (but please see above and [59]). We conclude this article with an overview of future research directions and opportunities to harness the regulated ERAD of native proteins as a therapeutic strategy for specific diseases.

## 1. Lipid Metabolism

The ER serves as a major hub of lipid metabolism. A variety of lipids, including fatty acids, triacylglycerol, phospholipids, and cholesterol, are synthesized in the ER. The lipids synthesized in the ER can be used by the cell or can be assembled into lipoprotein particles in the ER lumen, which are then transported to the circulatory system [60,61]. As a result, a variety of highly evolved mechanisms are employed to regulate these processes, since lipid imbalance can disrupt cellular homeostasis and cause several diseases, including dyslipidemia, insulin resistance (i.e., diabetes), fatty liver disease, and cardiovascular injury [62]. These regulatory processes alter substrate transcription, translation, enzyme kinetics, and/or degradation. Indeed, some of the earliest and best-described ERAD substrates are linked to lipid metabolism [63,64]. In this next section, we provide brief summaries of how select enzymes required for lipid homeostasis are regulated by ERAD.

### 1.1. Erg1/Squalene Monooxygenase

**Figure 1:**
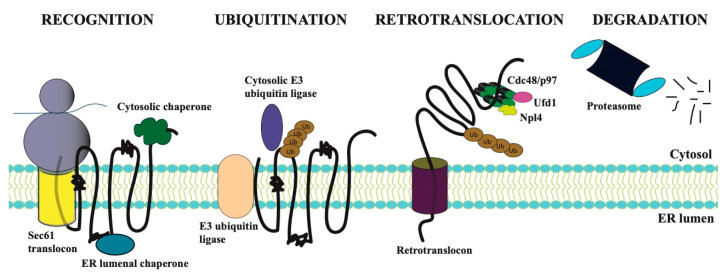
The endoplasmic reticulum-associated degradation (ERAD) pathway consists of four steps. The degradation pathway for a generic integral membrane protein in the ER is shown, and misfolded regions in the lumen, membrane, and cytosol are represented within the polypeptide chain as condensed regions. Recognition: Integral membrane proteins enter the ER concomitant with protein translation on ER-associated ribosomes and with the assistance of the Sec61 translocon. A misfold region in a nascent polypeptide is recognized by cytosolic or lumenal chaperones. Ubiquitination: The ubiquitination machinery—and more specifically an E3 ubiquitin ligases, often along with an E2 ubiquitin conjugating enzyme (not shown)—is next recruited to the misfolded protein, which is then conjugated with a polyubiquitin chain. The chain most commonly contains Lys-48 isopeptide linkages, and a minimum of four ubiquitins is required for proteasome-dependent degradation [14]. Retrotranslocation: The ubiquitinated protein is retrotranslocated through an ER-integrated retrotranslocon. Retrotranslocation requires ATP-dependent extraction mediated by the Cdc48 (in yeast) or p97 (in mammals) complex. The Cdc48/p97 complex also consists of two associated factors, Npl4 and Ufd1, which aid in ubiquitinated substrate capture. Degradation: During and/or after retrotranslocation, the misfolded ubiquitination substrate is degraded by the 26S proteasome into short peptide fragments.

**Figure 2:**
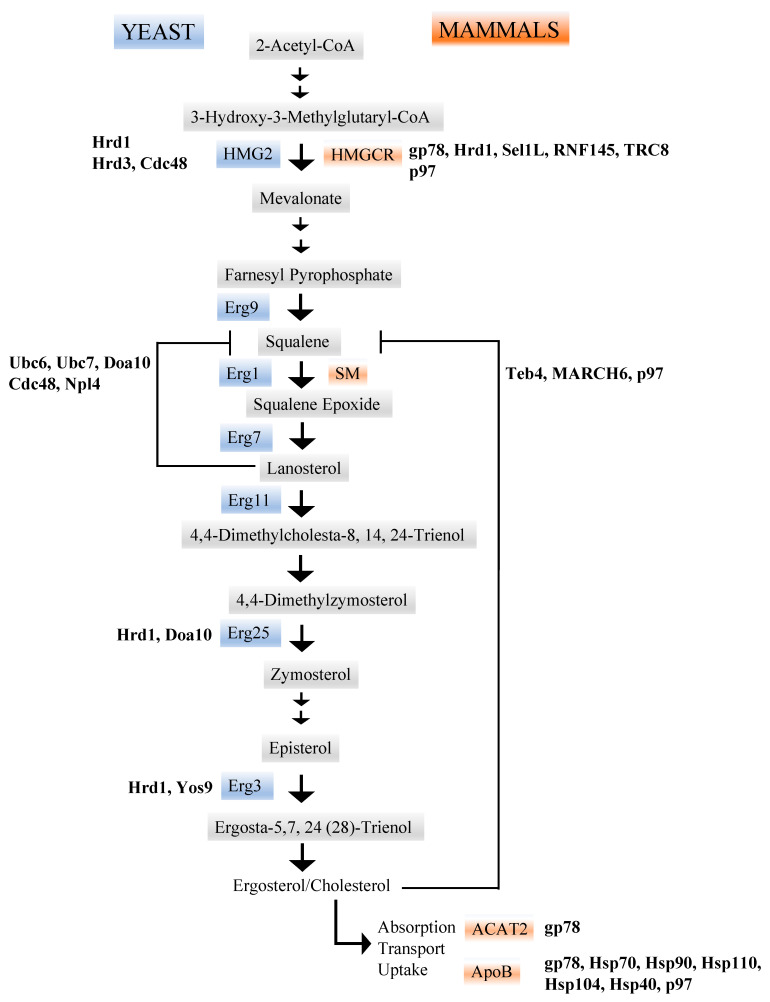
The biosynthetic pathway that leads to the generation of ergosterol (in yeast and fungi) and cholesterol (in higher organisms). The ergosterol and cholesterol biosynthetic pathways are catalyzed by a cascade of enzymes, including those shown as well as others that were not highlighted in the text. Select examples of feedback loops are also shown. Key enzymes regulated by ERAD in yeast (ergosterol biosynthetic pathway, in blue) and mammals (cholesterol biosynthetic pathway, in orange) are also noted. Two factors (ApoB and ACAT2) that act after cholesterol synthesis in mammals are also highlighted. Where studied, select examples of proteins that facilitate substrate degradation by the ERAD pathway and that act on specific enzymes in these pathways are displayed in bold.

Early work suggested that the application of proteomic technologies could be used to identify “native” ERAD substrates, i.e., wild-type proteins expressed at endogenous levels in cells that might be subject to ERAD-dependent degradation [65,66,67]. To expand upon this analysis and apply the technology to a model organism, a SILAC (stable isotope labeling by amino acids in cell culture)-based screen for substrates in yeast deleted for Doa10, an ERAD-associated E3 ubiquitin ligase, was performed. Among the identified hits was Erg1 [27,28]. Erg1, or squalene monooxygenase (SM), catalyzes the epoxidation of squalene to 2,3 oxidosqualene (squalene epoxide), which is the precursor for lanosterol (Figure 2). The identification of Erg1 as an ERAD substrate was validated by Erg1 stability measurements in cycloheximide chase experiments in yeast lacking Doa10. Interestingly an earlier proteomic analysis had identified Erg1 as a potential ERAD substrates in yeast lacking Npl4, a member of the Cdc48 complex that is responsible for retrotranslocation [28]. Indeed, Erg1 was also stabilized in yeast cells deleted for Ubc6 or Ubc7, which represent the two major ubiquitin conjugating enzymes (E2s) required for ERAD [27], as well as in strains containing a temperature-sensitive allele of *CDC48*, which encodes the protein (Cdc48, or p97 in mammals) required for ATP-dependent retrotranslocation (Figure 1). In contrast, defects in the Hrd1 complex, which represents the other primary E3 ubiquitin ligase complex in yeast [68,69], had no effect on Erg1 levels. Next, to investigate the critical lysine residues responsible for the Doa10-dependent degradation of Erg1, several Erg1-derivatives harboring individual or double lysine to arginine mutations were generated [27]. Cycloheximide chase experiments revealed that substitution at Lys-311 stabilized the protein, indicating that the Erg1 is targeted specifically for degradation. In other words, if the protein was globally misfolded, one might expect that numerous lysine residues might be modified.

Since several steps in sterol synthesis are regulated by feedback mechanisms (Figure 2), the sterol dependence of the Doa10-dependent degradation of Erg1 was examined [27]. Consistent with selective targeting, the treatment of cells with zaragozic acid, a small molecule inhibitor of Erg9, stabilized Erg1. An inhibitor of Erg7 that catalyzes the production of lanosterol also stabilized Erg1. In contrast, treatment of cells with fluconazole, which inhibits Erg11, accelerated Erg1 degradation, and this effect was absent in *doa10Δ* cells [27]. These data strongly suggest that lanosterol accumulation stimulates the Doa10-dependent degradation of Erg1. Consistent with this hypothesis, when the authors utilized shotgun lipidomics [70] to assess the lipid profile of cells lacking Doa10, the levels of ergosterol were reduced in *doa10Δ* cells by 13%, there was a five-fold increase in lanosterol, and the cells had 40% more sterol esters compared to wild-type yeast.

In mammals, the homolog of yeast Erg1 is squalene monooxygenase (SM, also see above and Figure 2). Early work indicated that the degradation of SM is proteasome-dependent and is triggered by increased cholesterol. This contrasts with the situation with Erg1, in which degradation is lanosterol-dependent (i.e., via an intermediate) [71]. Proteasome inhibition increased levels of ubiquitinated SM, which further increased upon treatment with cholesterol. Nevertheless, there is some conservation of the mechanisms controlling Erg1 and SM degradation. Most notably, Teb4, the mammalian homolog of Doa10, similarly ubiquitinates and degrades SM in HEK cells [27]. In addition to Teb4, another E3 ubiquitin ligase (MARCH6) associates with and contributes to the degradation of SM [25,30].

### 1.2. Erg3

Erg3 is a C-5 sterol desaturase that introduces a C-5(6) double bond into episterol, which is also an intermediate during ergosterol biosynthesis (Figure 2). A SILAC-based screen in yeast cells lacking the other ERAD-associated E3 ubiquitin ligase, Hrd1, revealed Erg3 as an endogenous ERAD substrate [29]. In this study, the yeast strain also lacked Ire1 to exclude compensatory effects that might result from induction of the unfolded protein response (UPR), a transcriptional program that protects against ER stress [72]. Interestingly, an earlier proteomics screen to detect all of the proteins in yeast conjugated to ubiquitin had also identified Erg3 [73]. Consistent with both proteomic analyses, Erg3 protein levels rose in cells lacking Hrd1, and cycloheximide chase experiments in yeast strains lacking Hrd1 (but not Doa10, see above) stabilized Erg3. Erg3 also immunoprecipitated with Hrd1, and this interaction increased when Ubc7, the ubiquitin conjugating enzyme for Hrd1, was deleted. Since Erg3 is glycosylated, the authors of this study also conducted pulse chase experiments in yeast lacking Yos9, which helped identify glycosylated ERAD substrates targeted to the Hrd1 complex. The deletion of Yos9 stabilized Erg3, but interestingly, the deletion of the asparagine in Erg3 that becomes glycosylated had no effect on degradation, suggesting that Hrd1-dependent degradation is glycan-independent.

### 1.3. Erg25

Erg25, or methylsterol monooxygenase, converts dimethylzymosterol to zymosterol (Figure 2). Hints that the enzyme was targeted for ERAD were provided by mass spec experiments in which the levels of Erg25 were found to increase in yeast lacking Hrd1, and Erg25 was isolated in an experiment developed to identify novel proteasome substrates [32,34]. In this later substrate capture experiment, yeast cells expressed an epitope-tagged proteasome subunit (Pre8) and simultaneously lacked a drug efflux pump (Pdr5). Therefore, the proteasome could be inhibited and proteasome-bound substrates captured using affinity matrices and mass spec analysis. Other Ergs were also identified as potential ERAD substrates based on this analysis.

Consistent with ERAD targeting, Erg25 also associated with the proteasome in co-immunoprecipitation experiments, and cycloheximide chase studies revealed that the levels of Erg25 and ubiquitinated Erg25 were magnified when the proteasome was inhibited [32]. In contrast to Erg1 and Erg3, above, the degradation of Erg25 required both Hrd1 and Doa10. Since these enzymes recognize distinct domains in ERAD substrates (ER lumen and membrane versus cytosolic regions, respectively) [68,69,73], these data suggested that the “decision” leading to Erg25 degradation requires the lipid-mediated destabilization of domains throughout the Erg25 protein. Consistent with regulated degradation, treatment with fluconazole, which inhibits Erg11 (see above), slowed Erg25 degradation. Erg25 was also stabilized in yeast lacking the downstream Erg2 and Erg3 enzymes (Figure 2), suggesting regulation by the accumulation of downstream intermediates.

### 1.4. Hydroxy-3-Methylglutaryl-Coenzyme A Reductase (HMGCR)

Both ergosterol (in yeast/fungi) and cholesterol (higher organisms) are synthesized from isoprene units via the mevalonate pathway, and ~20 steps/reactions are required from the starting material for this pathway, acetyl-CoA. In contrast to yeast, cholesterol can be acquired from the diet or it can be manufactured within cells. The ER-localized enzyme, HMGCR (Figure 2), catalyzes the production of mevalonate from HMG-CoA and represents the rate-limiting step in the production of both ergosterol and cholesterol, as well as in the synthesis of thousands of isoprene derivatives, including sterols, terpenes, bile salts, dolichol, heme A, and ubiquinone. Thus, HMGCR is subject to multiple levels of regulation, including transcription, translation, post-translational modifications, and degradation [74,75]. For example, when the levels of circulating cholesterol are low, the transcription of HMGCR increases, thereby inducing endogenous cholesterol synthesis. Combined with the fact that HMGCR is the target of statins, which represents the most effective way to lower circulating levels of cholesterol and other fats [61,75,76,77], and, based on the development of a powerful yeast genetic screen in which its biogenesis can be examined [64], HMGCR is one of the most widely studied substrates that is metabolically regulated by ERAD.

Early work established that the degradation of HMGCR is entirely via the proteasome and that vacuole or lysosomal inhibition in yeast or mammalian cells, respectively, has no effect on HMGCR levels [35,78,79]. Work in mammalian cells also indicated that one or potentially several E3 ubiquitin ligases, i.e., gp78, Hrd1, RNF145, and/or TRC8, regulate HMGCR [33,36,37,38,39]. The recruitment of the E3s is controlled by INSIG, which in turn senses whether a cholesterol precursor, 24,25-dihydroxysterol/25-hydroxycholesterol, is bound to HMGCR. The role of gp78 has been controversial and may reflect genetic variability, extracellular factors, or off-target effects. Several of these E3s might also act together or in sequence. Regardless, HMGCR is ultimately recognized by p97 (the Cdc48 homolog), retrotranslocated, and finally degraded by the proteasome [80,81].

In yeast, the degradation of Hmg2 (a regulated HMGCR ortholog) is strictly dependent on Hrd1 [15], and, like mammalian HMGCR, Hmg2 contains a sterol-sensing domain. When bound to an alternate cholesterol precursor, geranyl-geranylpyrophosphate (GGpp), the metabolite induces direct destabilizing conformational change in Hmg2, which triggers Hrd1- and Cdc48-dependent degradation [40]; a Hrd1 stabilizing protein, Hrd3, is also required for degradation. This regulatory event—allosteric misfolding—has been referred to as “mallostery”, and it is likely that other enzymes undergo a similar process in which substrates are delivered for efficient, controlled destruction. Along with differences in the lipid that regulates degradation (i.e., 25-hydroxycholesterol versus geranyl-geranylpyrophosphate), there is another interesting distinction from the situation in mammalian cells: the yeast INSIG homolog, Nsg1, does not recruit E3s but instead stabilizes Hmg2 in the absence of GGpp [82]. Together, while the details underlying some facets of regulated degradation differ, it is fascinating that the overall sterol regulatory pathway has been completely conserved during the evolution of eukaryotes.

### 1.5. Acetyl-CoA Acetyltransferase 2 (ACAT2)

ACAT2, expressed in the liver and small intestine, converts cholesterol and a free fatty acid to a cholesterol ester, thereby aiding cholesterol absorption and lipoprotein assembly. Elevated levels of ACAT2 have been linked to atherosclerosis, metabolic syndrome, and diabetes [83,84]. However, when lipids are deficient, ACAT2 is ubiquitinated and degraded [41].

Evidence supporting the targeting of ACAT2 to the ERAD pathway derived from several results. First, proteasome inhibition increased ACAT2 levels (Wang et al., 2017). Unexpectedly, a mutant in which all Lys residues were replaced with Arg was still ubiquitinated in the absence of added cholesterol. This observation encouraged the authors of this study to mutate unconventional ubiquitin-conjugated amino acids, e.g., Ser, Thr, and Cys. It was through this labor-intensive analysis that Cys-277 was uncovered as the position critical for ubiquitin-dependent degradation. Consistent with Cys modification, addition of beta-mercaptoethanol shifted the migration of ubiquitinated ACAT2 to its expected unmodified molecular weight. Although ubiquitin is usually conjugated onto Lys resides, non-canonical ubiquitination at Cys has been observed for several proteins, including Pex5 and Pex20, which contribute to protein translocation in the peroxisome [85,86,87]. Additional evidence supporting an ERAD-like targeting mechanism for ACAT2 identified gp78 as the E3 ubiquitin ligase that most likely modifies and catalyzes enzyme degradation.

Why would ACAT2 become ubiquitinated by an unconventional amino acid? The authors speculated that Cys can be easily oxidized, which might serve as an efficient regulatory switch [41]. In fact, treatment of cells with oxidizing agents (i.e., menadione and hydrogen peroxide) stabilized ACAT2. More relevant, high levels of lipids led to oxidative stress, and, in contrast to the situation when lipids were depleted, ACAT2 was stable when saturated fatty acids and cholesterol levels rose. Consequently, high concentrations of cholesterol, sterol intermediates (lanosterol), and saturated fatty acids can all generate reactive oxygen species that oxidize Cys-277 to a sulfenic acid (-SOH). This in turn stabilizes ACAT2 since the Cys can no longer be modified, thereby increasing the levels of cholesterol esters. The physiological relevance of this work was underscored by the fact that the expression of a Cys-to-Ala mutant at position 277 (which was more stable than that in wild-type mice) led to greater insulin-insensitivity. Overall, this discovery highlights how analyses of these unconventional ERAD substrates have increased our understanding of metabolic regulatory mechanisms, of the relationship of these mechanisms to human disease, and of the ubiquitin–proteasome pathway.

## 2. Other Substrates

In addition to the targeting of regulated enzymes linked to lipid homeostasis, proteins that are selected for ERAD when metabolic conditions change include substrates that perform myriad roles in the cell. These assorted factors are found in various organisms and are regulated in diverse ways. Yet, as above, the study of these substrates has further expanded our knowledge of how cellular proteostasis is impacted by changes in nutrient levels.

### 2.1. Apolipoprotein B (ApoB)

**Figure 3:**
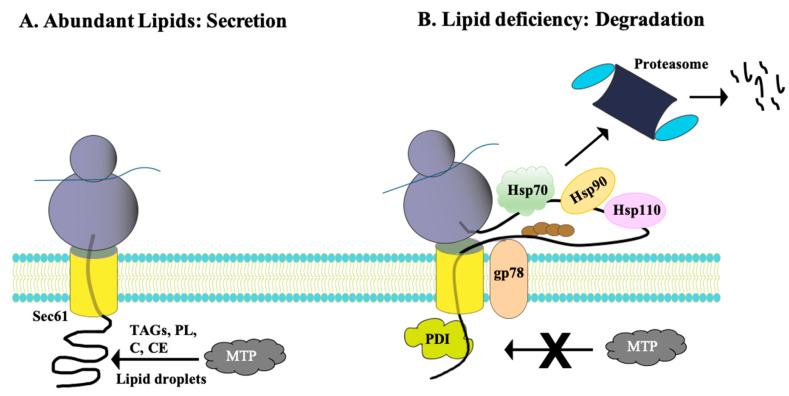
Early steps during apolipoprotein B (ApoB) biosynthesis. (**A**) Under lipid-rich conditions, cholesterol (C), cholesterol esters (CE), phospholipids (PL), and triacylglycerides (TAG) from the ER membrane and from ER lumenal lipid droplets assemble onto ApoB with the assistance of the MTP complex as it is co-translationally translocated through Sec61. (**B**) Under lipid-poor conditions, co-translational translocation through Sec61 slows but translation continues, depositing large polypeptide segments of ApoB in the cytoplasm. The depicted chaperones, as well as the gp78 ubiquitin ligase, associate with and facilitate ApoB targeting the proteasome.

ApoB is an unconventionally regulated ERAD substrate because it is co-translationally degraded [88,89]. As ApoB is translocated into the ER through the Sec61 translocon while still attached to the ribosome [89,90], the microsomal triglyceride transfer protein complex, MTP, delivers triacylglycerols, phospholipids, cholesterol, and cholesteryl esters from the ER membrane and ER lumenal lipid droplets onto ApoB (Figure 3A). These lipids are assembled onto so-called lipid associating domains in ApoB [91,92,93,94,95]. This lipid loading event is further facilitated by pause transfer sequences that are encoded within ApoB. More specifically, the sequences slow translocation so the lipids can bind more readily to β-sheet-rich stretches in the lipid associating domains in ApoB [96,97,98]. Once lipidated via these steps in the ER, the primordial particle is next transported to the Golgi complex for further lipidation. Ultimately, a lipoprotein particle is formed, one that become a very low-density lipoprotein (VLDL) particle or a chylomicron in the liver or intestine. These particles are secreted into the circulatory and lymphatic systems [99,100,101].

In contrast to this situation, when lipids are deficient, ApoB translocation slows. However, continued translation exposes large hydrophobic loops to the cytosol where molecular chaperones and the ubiquitination machinery target the protein for degradation [102] (Figure 3B). The chaperones that associate with ApoB include the ER lumenal lectin, calnexin, an ER membrane-associated Hsp40 homolog (p58^IPK^), as well as the lumenal factors protein disulfide isomerase (PDI) and cytosolic Hsp70, Hsp90, Hsp110, and—in a yeast model—Hsp104, which is a protein disaggregase [103] (Table 1). Whereas some of these proteins promote the degradation of ApoB (Hsp70, Hsp90, the PDIs, Hsp104), Hsp110 stabilizes the protein [42,43,44,45,46,47,48,49,50,51]. The identification of these factors was made possible by utilizing yeast, hepatoma cell lines, and in vitro experiments in which ApoB biogenesis could be reconstituted.

After selection by specific chaperones, ApoB is ubiquitinated by gp78 in humans [104,105]. The protein is then degraded by the 26S proteasome (Figure 3B). Because of the importance of regulating the levels of circulating cholesterol and other lipids, current efforts are targeting ApoB to treat some cases of familial hypercholesterolemia that are resistant to the commonly prescribed statins, which directly inhibit HMGCR [106,107,108] (also see above and Discussion). More generally, it is stunning that this ~500 kDa protein is constitutively targeted for ERAD between meals, suggesting that the importance of regulated degradation outweighs the energetic cost of synthesizing this large, labile protein.

### 2.2. Inositol 1,4,5-Triphosphate Receptors (IP_3_R)

IP_3_ receptors are tetrameric ER transmembrane channels responsible for the release of stored calcium upon binding to the second messenger, IP_3_, which is generated in the plasma membrane in response to various signal transduction pathways [109]. IP_3_ receptors are conserved among many species and are expressed in all animal tissues. They play key roles in several processes that are dependent on calcium signaling, including cell division, apoptosis, fertilization, development, and memory [110,111]. Thus, disruption in IP_3_ receptor function can cause a plethora of diseases, including ataxia, neuronal dysregulation, and cardiovascular diseases [112]. Interestingly, elevated levels of IP_3_ resulting from treatment with certain activators leads to receptor ubiquitination and proteasome-dependent degradation [54,55,113]. Because IP_3_ receptor degradation is independent of treatment with brefeldin A, which blocks ER-to-Golgi and thus lysosome transport, and is resistant to lysosomal protease inhibitors, the receptors are degraded at the site in which they act, i.e., in the ER [109,114,115,116]. These data position the ERAD pathway as a means to downregulate receptor activation.

Consistent with this view, the ERAD-associated E2 ubiquitin conjugating enzyme Ubc7 mediates receptor ubiquitination, and degradation requires the p97 complex [52,56]. Other factors required for IP_3_ degradation include two other transmembrane proteins in the ER, SPFH1 and SPFH2, which encode SPFH (stomatin, prohibitin, flotillin, and HflC/K) domains [54,55]. SPFH1 and SPFH2 localize to cholesterol-rich regions in the ER membrane and are thought to recognize the lumenal regions of activated IP_3_ receptors. These proteins might then recruit Ubc7 as well as an E3 ubiquitin ligase. To date, the only E3 known to ubiquitinate IP_3_ receptors is the ER membrane localized ubiquitin ligase RNF170 [53]. Overall, like ApoB, this regulated form of ERAD provides a robust mechanism to rapidly downregulate a transient metabolic signal/new metabolic state.

### 2.3. Pca1

Pca1 is a transmembrane heavy metal (cadmium) P-type ATPase transporter in the yeast *Saccharomyces cerevisiae* and is expressed at the plasma membrane [117,118,119]. In the absence of cadmium in the growth media, Pca1 is degraded by the ERAD pathway [57]. Interestingly, ERAD targeting is dependent on the Pca1 N-terminal domain, which acts as a cadmium sensor. More specifically, cadmium binds to Cys residues in this domain, thereby masking a degradation signal. A genetic screen using a GFP-tagged form of the transporter revealed that Pca1-GFP was stabilized when the gene encoding Cue1, which recruits Ubc7 to the ER membrane [120], was deleted. Pca1 was also stabilized in yeast lacking Doa10, which was further validated by observing decreased ubiquitination levels when Doa10 was absent. Moreover, chemical crosslinking revealed association between the N-terminal domain of Pca1 and Doa10. Interestingly, the N-terminal degron becomes resistant to proteolysis in the presence of cadmium, suggesting that the metal induces a major conformational change in the transporter. This mechanism is reminiscent of the regulated degradation observed for HMGCR in yeast (see above). In both cases, a specific ligand/effector significantly and directly regulates protein structure, thus altering substrate stability.

### 2.4. β-catenin

The Wnt/β-catenin signaling pathways play critical roles in hematopoietic stem cell renewal, cell proliferation, and cellular differentiation. Based on its importance, defects in Wnt/β-catenin signaling result in multiple pathologies, including several cancers, Alzheimer’s disease, cardiovascular diseases, and schizophrenia [121,122,123]. β-catenin serves as the cellular effector protein of this pathway, and the levels of this molecule are regulated by negative feedback mechanisms [124]. In turn, Wnt is a lipid-modified secreted protein whose transport/secretion from the cell requires a membrane protein, Evi/Wls, that was defined in several genetic screens [125,126,127]. Interestingly, the level of Evi/Wls is regulated by the ERAD pathway, and the post-translational modification of Wnt controls Evi/Wls stability [128]. In a subsequent study, an RNAi-based screen was use to establish that Evi/Wls quantity control is orchestrated by a consortium of ERAD-associated factors [129].

Wnt signaling is also controlled in another manner that is linked to the ERAD pathway. A recent forward genetics screen in mice revealed that mutations in the limb region1-like gene (LMBR1L) led to impaired lymphocyte development [58]. LMBR1L-interacting proteins were then identified by co-immunoprecipitation and mass spec analysis. Included amongst the LMBR1L partners were β-catenin and other Wnt signaling components (ZNRF3, LRP6, glycogen synthase kinase–3α (GSK-3α), and GSK-3β), as might be expected. More surprisingly, several factors linked to the ERAD pathway were also identified. These included p97, which as noted above drives substrate retrotranslocation, and gp78, an E3 ubiquitin ligase (see above), which when overexpressed increased β-catenin ubiquitination. In addition, a protein with a ubiquitin association domain, UBAC2, and a ubiquitin-like UBX domain-containing protein, UBXD8, were both identified. UBAC2 and UBXD8 appear to associate with one another and have also been linked to ERAD [130,131,132]. These data suggest that LMBR1L may be a member of both the Wnt/β-catenin signaling and ERAD pathways.

The canonical deactivation of β-catenin signaling is caused by a destruction complex comprising GSK-3α and GSK-3β, scaffolding proteins Axin1 and DVL2, and the β-TrCP E3 ubiquitin ligase [133,134]. Interestingly, lymphocytes lacking LMBR1L exhibited decreased levels of all of the proteins of this destruction complex, further implicating LMBR1L as a negative regulator of Wnt/β-catenin signaling. The homozygous knockout of LMBR1L in mice led to higher levels of β-catenin as well as to a Wnt receptor, FZD6, and a co-receptor, LRP6. Additionally, homozygous knockout of gp78 in mice displayed increased levels of β-catenin, FZD6, and LRP6 in the ER [58]. Collectively, these data indicate that LMBR1L is a component of an ERAD-associated E3 ubiquitin ligase complex that stimulates the degradation of factors required for Wnt/β-catenin signaling. In the future, it will be exciting to determine whether this complex is required for the ERAD of other substrates.

## 3. Conclusions

In this review, we have outlined the properties of select, functioning protein substrates that are targeted for regulated degradation via an ERAD-like pathway. It is likely that the identities of other substrates in this category will emerge. As noted throughout the article, an examination of these non-canonical ERAD substrates led to the discovery of new principles in the ERAD field. Furthermore, it is not a coincidence that all of these substrates are either integral membrane proteins or—during their lifetime—must associate with the ER membrane. In fact, the ER bilayer provides an ideal platform to concentrate the many enzymes needed to drive the selection and ubiquitin- and proteasome-dependent degradation of these and other substrates. It is noteworthy, then, that some misfolded cytosolic proteins are targeted to the ER membrane for degradation [135]. Together, a better understanding of this recruitment process and an expansion of the number of proteins targeted for ERAD represent important future undertakings.

In parallel to these efforts, it is also vital to understand how the regulated selection and degradation of folded/functional proteins occurs at the molecular level, especially with regard to enzymes that control lipid metabolism [136]. One example provides a hint at how this might occur and is exemplified by factors in the OLE pathway in yeast, which is driven by the proteasome-dependent clipping of ER-tethered transcription factors and extraction by the Cdc48 complex [137,138]. Since this pathway controls the levels of unsaturated lipids in the ER membrane, one hypothesis is that the conformations of the transcription factors—Mga2 and Spt23—that regulate the production of enzymes required for fatty acid desaturation change in response to membrane fluidity. This hypothesis was supported upon the demonstration that two transmembrane-spanning regions in Mga2 rotate relative to one another when the levels of unsaturated fatty acids are low [139]. This resulting conformational change next initiates Mga2 ubiquitination by an E3 ligase, Rsp5, that is rarely used for ERAD [140]. Based on this elegant study, it is likely that changes in transmembrane domains relative to one another are similarly used to drive the regulated degradation of other substrates.

Another example of the molecular underpinnings of regulated ERAD substrate selection derives from studies on Hmg2 in yeast. Data from Hampton and colleagues [141] suggested that the exposure of hydrophilic residues within the transmembrane domain of Hmg2 leads to the identification of this substrate by the Hrd1 ubiquitin ligase. Again, it is also likely that similarly induced conformational changes in other wild-type proteins might be sufficient to support regulated destruction via ERAD.

Based on the importance of controlling lipid metabolism as a means to treat disease, there is of course significant interest in the development of drugs that alter the ERAD of native substrates. For example, statins are the first-line treatment for individuals with high levels of circulating cholesterol, and, as noted above, the drug targets HMGCR. Statins have been in wide use for >35 years, but they also inhibit the production of cholesterol derivatives and compounds built from isoprenes. In addition, not all patients respond equally to statins, and the decrease in circulating low-density lipoproteins (LDLs), which carry the vast majority of cholesterol between meals, can vary from 5-70% in an individual [142,143]. This variation is attributed to polymorphisms in genes linked to statin metabolism (i.e., pharmacodynamics and pharmacokinetics) [144,145,146,147]. To overcome these hurdles, other treatments have been developed that either directly target ApoB via antisense oligonucleotides or reduce LDL receptor levels, but these can elicit side effects, are costly, and/or have not been approved for all patients due to these limitations [148]. Based on these issues, there is clearly a need to modify other enzymes in the cholesterol biosynthetic pathway, and a continued molecular definition of how these and other regulated enzymes that are targeted for ERAD is clearly warranted.

## Figures and Tables

**Table 1:** Select examples of natively folded ERAD substrates.

Organism	Substrate	Function	ERAD Effectors	References
Yeast/Mammals	Erg1/Squalene Monooxygenase (SM)	Ergosterol/Cholesterol synthesis	Ubc6, Ubc7, Doa10/TEB4, MARCH6, Cdc48/p97, Npl4	[25,26,27,28,29]
Yeast	Erg3	Ergosterol synthesis	Hrd1, Yos9	[30,31]
Yeast	Erg25	Ergosterol synthesis	Hrd1, Doa10	[32,33]
Yeast/Mammals	3-Hydroxy-3-Methylglutaryl-CoA Reductase (HMGCR)	Sterol synthesis	Hrd1, Hrd3/Sel1L, gp78, TRC8, RNF145, Cdc48/p97,	[33,34,35,36,37,38,39,40]
Mammals	Acetyl-CoA Acetyltransferase 2 (ACAT2)	TAG synthesis Cholesterol	gp78	[41]
Yeast/Mammals	Apolipoprotein B (ApoB)	Lipoprotein assembly, transport, uptake	Hsp40, Hsp70, Hsp90, Hsp110, Hsp104, PDI, gp78, p97	[42,43,44,45,46,47,48,49,50,51]
Mammals	IP_3_ receptor	Calcium signaling	Spfh1, Ubc7, gp78, RNF170, p97	[52,53,54,55,56]
Yeast	Pca1	Cadmium transporter	Ubc6, Doa10, Cdc48	[57]
Mammals	β-catenin	Hematopoietic stem cell renewal, survival and differentiation	UBAC2, UBXD8, gp78	[58]

## Data Availability

All data are available upon request.
